# Mini-Review: Antibiotic-Resistant *Escherichia coli* from Farm Animal-Associated Sources

**DOI:** 10.3390/antibiotics11111535

**Published:** 2022-11-02

**Authors:** Chunming Xu, Lingqiang Kong, Yonghong Liao, Yuan Tian, Qi Wu, Haosi Liu, Xiumin Wang

**Affiliations:** 1 School of Light Industry, Beijing Technology and Business University, Beijing 100048, China; 2 Key Laboratory of Cleaner Production and Integrated Resource Utilization of China National Light Industry, Beijing Technology and Business University, Beijing 100048, China; 3 Key Laboratory of Feed Biotechnology, Ministry of Agriculture and Rural Affairs, Beijing 100081, China; 4 Institute of Feed Research, Chinese Academy of Agricultural Sciences, Beijing 100081, China

**Keywords:** antibiotic resistance, *Escherichia coli*, farm animals, alternatives, disinfection

## Abstract

*Escherichia coli* is one of the most frequent causes of gastro-intestinal and extra-intestinal diseases in animals and humans. Due to overuse and misuse of antibiotics, recent years have seen a rapidly increasing prevalence of antibiotic-resistant (AR) *Escherichia coli* globally; particularly, AR *E. coli* from farm animal-associated sources and its antibiotic resistance genes (ARGs) are becoming a global concern, with clinical negative effects on both human and animal health. The aim of this review was to explore the prevalence trends of AR *E. coli* from farm animals, waste treatment, and aquatic environments. The disinfection methods of AR *E. coli* and possible alternatives to antibiotics were also highlighted. The current review highlights that the prevalence of AR *E. coli* from food animals, products, and animal waste is increasing at an alarming rate, but is reduced at waste treatment plants. Ultraviolet (UV) treatment, surface plasma oxidation, and biochar are commonly used to effectively eliminate AR *E. coli*. Some probiotics, plant extracts, and antimicrobial peptides (AMPs) are arousing interest as promising alternatives to antibiotics to fight against AR *E. coli*. The current review suggests that AR *E. coli* from farm animal-associated sources is prevalent and poses a serious global threat to public health. This review provides an avenue for further research, development, and application of novel strategies to minimize antibiotic resistance in *E. coli* of farm animal origin.

## 1. Introduction

*Escherichia coli* is found in the environment, food, and the guts of animals and humans. It is an opportunistic pathogen that can cause gastro-intestinal and extra-intestinal diseases, such as diarrhea, enteritis, bacteremia, urinary tract infection, and other infections in animals and humans [1,2,3,4,5]. *E. coli* is also found in a major portion of feces from animals [6,7]. Due to overuse and misuse of antibiotics, *E. coli* of animal origin displays resistance against various antibiotics, including tetracyclines, aminoglycosides, β-lactams, fluoroquinolones, third-generation cephalosporins, etc. [8,9,10,11]. It has been demonstrated that more than 90% of *E. coli* isolated from food animals (including healthy broiler chickens, cattle, and pigs) in Korea during 2010–2020 exhibited high resistance to quinolones and cephalosporins [12]. Meanwhile, antibiotic-resistant (AR) *E. coli* that carries antibiotic resistance genes (ARGs) is also present in multiple hosts and environmental compartments as a normal inhabitant or a temporary or persistent colonizer, and can be globally transmitted between animals and humans [13]. In some waste treatment plants and aquatic environments, AR *E. coli* concentrations were up to 10–10^5^ CFU/mL, higher than the requirement for irrigation use of water (10 CFU/100 mL) [14,15,16], posing a serious threat to public health [3,4,17,18,19].

The dissemination of AR *E. coli* and its ARGs is considered to occur frequently in farm animal-associated conditions [20,21]. Animals and products serve as potential reservoirs and vectors for the spread of AR *E. coli*. AR *E. coli* that carries ARGs (such as *bla_CTX-M_*, *sul1*, *tetA*, *tetB*, etc.) on livestock farms can transfer into the surrounding environment, such as water bodies, soils, etc. [1,20,22,23]. Moreover, AR *E. coli* and its ARGs of animal origin can also be transferred to other bacteria through horizontal gene transfer (HGT), which plays a key role in the acquisition, accumulation, and dissemination of ARGs in bacteria [24,25,26,27]. Some ARGs (including *sul3*, *cmLA*, *aadA1*, *aadA2*, *tetR*, *tetA*, and *dhrfI*) from a commensal *E. coli* strain isolated from food animals such as broilers and pigs could be transferred to humans [28,29]. Noticeably, multi-drug resistant (MDR) *E. coli* isolates from livestock are identical to those of human-origin isolates, indicating high transferability of AR *E. coli* [26]. AR *E. coli* and its ARGs pose serious risks to animal and human health [5], requiring effective strategies to combat them.

To fight against AR *E. coli* of animal origin, some traditional and novel strategies, including ultraviolet (UV) treatment, surface plasma oxidation, and biochar, have been reported to effectively kill AR *E. coli* by inhibiting DNA replication and destroying the cell membrane [30,31,32]. A few ARGs (including *tetC*, *tetW*, *bla_TEM-1_*, *ampC*, etc.) of *E. coli* of animal origin were remarkably removed or reduced after plasma or biochar oxidation treatment, and conjugative transfer of ARGs (such as integron gene-*intI1*) was dramatically inhibited [31,33]. The advantage of using these methods is that they display high efficiency, simple equipment, and no/low exogenous chemical residues [30,33,34]. It is worth mentioning that recently, some potential alternatives to antibiotics, such as probiotics, plant extracts, antimicrobial peptides (AMPs), etc., are emerging to address the emergence and spread of AR *E. coli* due to their potent biological functions, with health benefits [35,36,37,38]. Especially for AMPs, a class of effector molecules with less than 100 amino acids, are found in almost all living organisms and display potent antibacterial activity against AR bacteria (ARB) and low risk of the development of drug resistance [39,40]. Hitherto, several AMPs (such as microcin, magainin, cathelicidin, etc.) are regarded as a promising class of antimicrobial agents to overcome AR *E. coli* [40,41,42,43,44].

**Figure 1:**
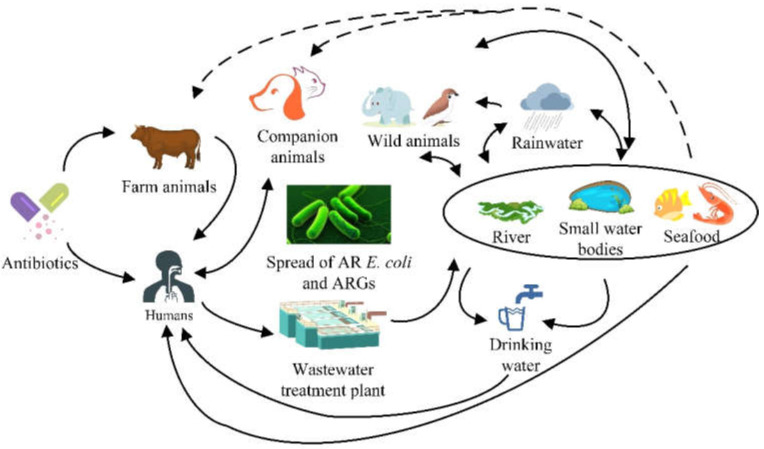
Multiple pathways involved in the transfer of AR *E. coli* and its ARGs in animals, humans, and the environment. Antibiotics used in farm animals may lead to AR *E. coli*, which is present in food products, feces, manure, waste, treatment plants, etc. AR *E. coli* and its ARGs can be transferred from farm animals to the surrounding environment, other animals, or even humans by multiple routes (including foods, waste, surface water, river, rainwater, drinking systems, etc.).

In this review, we introduce a brief outline of the current situation of AR *E. coli* from farm animal-associated sources (including farm animals, sewage/manure treatments, and aquatic environments), as well as strategies in the battle against AR *E. coli*. The possible routes of transmission of AR *E. coli* and its ARGs are summarized in Figure 1. By studying current AR *E. coli* of animal origin, we hope that more people can clearly understand its adverse effects, which may help develop more efficient strategies to fight against bacterial resistance from farm animal-associated sources.

## 2. Prevalence of AR *E. coli* from Farm Animal-Associated Sources

### 2.1. AR E. coli from Farm Animals

#### 2.1.1. Food Animals and Products

Animals may serve as potential reservoirs and vectors for the dissemination of ARB, including *E. coli*, which is a growing concern worldwide. Monitoring AR *E. coli* from food animals and products is vital to determine the emergence of antibiotic resistance and its associated risk to humans [20,45,46,47].

Avian pathogenic *E. coli*, which can cause avian colibacillosis and is common in Germany, Egypt, Pakistan, etc., displays high levels of resistance to ampicillin, tetracycline, ciprofloxacin, and other antibiotics [48,49,50]. Colistin-resistant *E. coli* and the *mcr-1* gene were found in broilers in Germany [49], posing a threat to human health by potential zoonoses [51]. A high prevalence of AR *E. coli* (100%), especially against tetracycline, gentamicin, colistin, imipenem, and β-lactams, has been observed in different poultry products and in poultry meat from different types of retailers; the majority of resistant genes were *tetA* and *bla_TEM_* [10]. Aklilu et al. analyzed broilers and retail chickens in food animals in Malaysia and found that 37.5% (27/72) of *E. coli* was positive for at least one antibiotic-resistant gene [52]. The prevalence was even more severe in Vietnam; colistin-resistant *E. coli* was found in about half of chicken samples, and most of AR *E. coli* contained the *mcr-1* gene [53]. Abdallah et al. analyzed retail lamb samples from Zagazig, Egypt, and found AR *E. coli* in nearly one-fifth of the samples [54].

Colistin-resistant *E. coli* was isolated from 78% (51/65) of raw beef and 53% (24/45) of ready-to-eat beef products in Egypt, with individual transmission in cattle [55]. Ahmad et al. surveyed raw milk samples from farms, milk vendors, and shops in the Peshawar district of Pakistan for MDR *E coli*. Among 28 isolated *E. coli*, six isolates were MDR *E. coli* and all identified *bla_CTXM_* resistance genes [56]. Furthermore, in recent years (2016–), AR *E. coli* was also found in ready-to-eat street foods in small and roadside outlets, which is a significant risk for human health (Table 1) [4,57]. Some 16.9–72.9% of *E. coli* in ready-to-eat street food (containing chicken) was resistant to cefepime, cefotaxime, imipenem, and meropenem; the most prevalent ARGs included *bla_TEM_* (40.68%), *bla_CTX_* (32.20%), *bla_SHV_* (10.17%), and *bla_NDM_*, indicating a potential risk of transmission from food to humans [4]. Meanwhile, AR *E. coli* was found in fresh seafood such as fish and shellfish sold in a retail market in Mumbai, India [3]. Some 71.58–95% of *E. coli* isolates were resistant to β-lactams and cephalosporins (including cefotaxime, cefpodoxime, and ceftazidime), with *bla_CTX-M_* (62.37%), *bla_SHV_* (23.35%), *bla_TEM_* (2.6%), *bla_OXA_* (7.06%), *bla_NDM_* (4.42%), and *bla_VIM_* (0.88%), indicating a high risk of transmission of MDR *E. coli* in seafood consumers and handlers [3]. Shellfish in marine environments were affected by effluents including rivers, domestic effluents, and sewage treatment plants that carry AR *E. coli* [58].

Therefore, the use of antibiotics in food animals has resulted in the selection of drug-resistant bacteria across the farm-to-fork continuum. It is essential to improve sanitary conditions and take intersectoral actions coordinated by the different organizations involved.

#### 2.1.2. Animal Waste

Animal waste is a large pool of ARB containing *E. coli* and its ARGs (Table 2), leading to the dissemination of AR *E. coli* and its ARGs into the environment, representing an important and dangerous environmental pollutant [59].

An investigation of *E. coli* isolated from the feces of native South African herbivores (including wildebeest, zebra, and giraffe), pets, and farm pigs was performed to analyze dissemination patterns of drug resistance [61]. The results suggested that herbivores native to South Africa may be important carriers in the transmission of AR *E. coli* [61]. The occurrence of integrons in AR *E. coli* strains isolated from free-grazing food animals (such as chickens, piglets, and yaks) in China was firstly determined by Rehman et al. [62]. A total of 432 *E. coli* strains isolated from free-ranging food animals in China were resistant to at least one class of antibiotics, including ampicillin, ceftriaxone, chloramphenicol, gentamicin, streptomycin, sulfonamide, tetracycline, etc. Integrons were detected in 6% of AR *E. coli* strains, indicating that precautionary measures are invoked to prevent the transfer of AR *E. coli* [62]. Weiss et al. analyzed a total of 1685 *E. coli* fecal samples from domestic animals, humans, and wild primates in western Uganda, and found that 499 *E. coli* isolates were resistant to 11 antibiotics tested [66]. The frequency of resistance was 57.4%, 19.5%, and 16.3% in *E. coli* isolates from people, domestic animals, and wild primates, respectively. The percentage of AR *E. coli* decreased with increasing local price of antibiotics. Moreover, 33.2% of resistant isolates with class 1 integrons were widely distributed in *E. coli* strains from different host species [66]. In Germany, researchers analyzed feces from a variety of animals, including ducks and pigs, and found that quinolone-resistant *E. coli* was widespread in livestock and food [64]. One survey from 2010 to 2020 showed that more than 90% of *E. coli* isolated from the feces of healthy broiler chickens, cattle, and pigs in Korea exhibited high resistance to quinolones and cephalosporins [12]. Additionally, AR *E. coli*, including cefazolin-resistant (43.1%), fluoroquinolone-resistant (22.1%), and β-lactam-resistant ones (9.4%), were found in dog feces, indicating the possible transmission of drug resistance in companion animals [2].

The epidemiology of AR *E. coli* in intensively produced poultry was investigated in South Africa by McIver et al. [23]. The results showed that 67.3% of *E. coli* isolates from the poultry industry were resistant to ampicillin (48.1%), tetracycline (27.4%), nalidixic acid (20.3%), trimethoprim–sulfamethoxazole (13.9%), and chloramphenicol (11.7%), which was similar to the antibiotics used in the poultry industry. The most frequently detected ARGs were *bla_CTX-M_* (100%), *sul1* (80%), *tetA* (77%), and *tetB* (71%). It indicated that intensive poultry farming may be a reservoir and a potential vehicle for the transmission of bacterial antibiotic resistance; some prompt measures must be taken to reduce the spread of bacterial resistance from the poultry industry to humans [23]. AR *E. coli* and *Salmonella* with resistance to third-generation cephalosporins or quinolones were detected in feces from cattle, goats, pigs, and poultry in Rwanda’s eastern province [11]. Suzuki et al. studied cow feces in animal farms and found that in farms where antibiotics are used carefully, AR *E. coli* has less impact on the surrounding wildlife and environment [65].

The percentage of AR *E. coli* in wastewater from adult cattle and veal calf slaughterhouses ranged from 5% to 87.5%; approximately 1,010 strains of MDR *E. coli* were detected to be released into contaminated rivers every day [60]. One pathogenic *E. coli* O157:H7 strain was transmitted into the environment by land application [60]. The prevalence of AR *E. coli* and antibiotic residues in open pig farm systems were analyzed by Wandee et al. [63]. They observed high levels of AR *E. coli* populations in open pig farm systems, probably due to antibiotic contamination in the water supply and additional application of antibiotics, such as neomycin or colistin, having a significant impact on the prevalence of AR *E. coli* in pig manure [63]. This suggests that more appropriate waste management guidelines should be proposed to reduce the spread of pathogenic MDR *E. coli* and its ARGs.

Furthermore, mismanagement, such as the continuous use of antibiotics in feed or the accumulation of antibiotics in meat duck deep litter, may also be responsible for the evolution of bacterial resistance [8]. *E. coli* isolates from duck farms and meat deep litter were resistant to various antibiotics, including tetracycline, ampicillin, doxycycline, ofloxacin, gentamicin, etc., indicating a high prevalence of multi-antibiotic resistance in *E. coli*. Deep litter is considered an ideal environment for the evolution of bacterial resistance [8].

### 2.2. AR E. coli in Waste Treatment Plants

Waste treatment plants commonly contain antibiotics, biocides, metals, and diverse microorganisms, being potential hotspots for ARB and their ARGs [67]. Approximately 10^3^–10^5^ CFU/mL AR *E. coli* was found in wastewater treatment plants (Table 3) [14,68,69], and a variety of drug resistance genes and virulence genes were detected [70]. Thus, the prevalence of AR *E. coli* and its associated ARGs in wastewater cannot be ignored.

In a survey of a city’s sewage treatment plants, the numbers of ampicillin- and chloramphenicol-resistant *E. coli* in municipal wastewater were up to 3 × 10^4^ CFU/mL, but greatly reduced (<10 CFU/100 mL) after treatment with peracetic acid (Table 3) [71]. In Norwegian treatment plants, all *E. coli*, including AR *E. coli*, were completely removed from wastewater by using ultrafiltration (UF) and nanofiltration (NF) membranes [67]. AR *E. coli* in municipal wastewater and river water was identified by Kaźmierczak et al. [68]; approximately 99.9% of AR *E. coli* was removed from wastewater treatment plants. However, AR *E. coli* isolates were at least one order of magnitude lower in summer than in winter. Residual resistant bacteria were up to 10^3^–10^5^ CFU/mL in treated wastewater, indicating a high spread risk of ARB in the environment. It revealed that despite the high efficiency of bacterial removal in wastewater treatment processes, considerable amounts of ARB are released into the environment with treated wastewater, and the percentage of ARB in total bacterial counts increases after wastewater treatment [68]. Yuan et al. found 35 MDR *Escherichia spp*. from animal farms, hospitals, and municipal wastewater treatment plants [72]. Each *Escherichia* isolate carried 21–26 ARGs and 8–12 mobile genetic elements (MGEs), and the isolates from livestock manure and wastewater treatment plants had greater diversity in plasmid profiles than hospital wastewater; more gene cassettes were also found in *Escherichia* isolates from livestock manure, which is possibly related to a higher occurrence of residual antibiotics or heavy metals [72].

The residual bacteria were less than 10 CFU/100 mL, which was compatible with the irrigation application of treated water. *E. coli* isolates with resistance to neomycin, florfenicol, amoxicillin, chlortetracycline, and sulfamethoxazole were found in pig farming (Table 3) [63]. Higher levels of *E. coli* and antibiotic residues were present in open farming systems than in closed systems, and there was no AR *E. coli* in the original excreta and wastewater. Meanwhile, an increased prevalence of AR *E. coli* was found in the sludge of anaerobic digestion and the waste stabilization pond [63]. Summerlin et al. (2021) found that the concentrations of ampicillin- and cephalothin-resistant *E. coli* in wastewater treatment plants were 2.5 ± 0.6–2.6 ± 2.0 log CFU/100 mL, higher than that of irrigation water (126 CFU/100 mL), posing potential health risks [73].

**Table 3:** AR *E. coli* and its ARGs in waste treatment plants (2016–2021).

Resistance to Antibiotics	ARGs	Contents of AR *E. coli*	Sources	Location	References
Ampicillin and chloramphenicol	*bla* and *cat*	3 × 10^4^ CFU/mL	Municipal wastewater	Italy	[71]
Ampicillin and trimethoprim–sulfamethoxazole	NN	NN	Influent wastewater	Norway	[67]
β-lactams and tetracycline	NN	1.25 × 10^5^ CFU/mL in winter and 1.25 × 10^3^ CFU/mL in summer	Upstream and downstream from the effluent discharge point	Poland	[68]
Aminoglycosides, sulfonamides, and quinolones	*aac-Ib*, *aacC2*, *aadA1*, *bla_CTX-M_*, *oqxB*, *qnrS*, *sul1*, *sul2*, *dfrA7*, *tetA*, and *tetG*	NN	Municipal wastewater treatment plants	China	[72]
Trimethoprim/sulfamethoxazole and tetracycline	*bla_CTX-M-1_*, *bla_TEM_*, *tetA*, and *tetB*	1.49–2.11 × 10^5^ CFU/ml	Urban wastewater treatment facility	America	[69]
Ampicillin, nalidixic acid, tetracycline, cotrimoxazole, and streptomycin	*Cit*, *Int1*, *Tn3*, *CTX-M1*, *IMP*, and *qnrS*	NN	Municipal and animal wastewater	Slovakia	[70]
Neomycin, florfenicol, norfloxacin, amoxicillin, colistin, chlortetracycline, and sulfamethoxazole	NN	3.0 × 10^3^–2.1 × 10^5^ CFU/mL	Aerobic digestion and waste stabilization pond	Thailand	[63]

NN: no data.

### 2.3. AR E. coli in the Aquatic Environment

At present, the rising emergence of AR and MDR *E. coli* is ubiquitous in the aquatic environment, which is a great concern for animal and human health [74]. Among them, the transfer effect of HGT has a significant influence on the spread of antibiotic resistance [75]. Hamelin et al. detected ARGs in *E. coli* isolates from different surface water areas within the St. Clair River and Detroit River by the DNA microarray method [76]. It was found that 48% of *E. coli* isolates carrying at least one ARG were downstream of wastewater effluent outfalls in an urban site, higher than that of other sites (24%). This suggests that AR *E. coli* from municipal wastewater may be widely transmitted in aquatic ecosystems [76]. The horizontal spread of MDR *E. coli* in small water bodies such as streams was mainly caused by runoff and leaching, which affect adjacent water bodies or large water bodies. During a rainfall period with grazing, the percentages of AR *E. coli* increased to 30–35%, higher than that of a dry period for grazing (<7%). Both chloramphenicol and tetracycline were most often found in water. Additionally, MDR *E. coli*, even with resistance to eight different antibiotics, accounted for 23% of the total resistant isolates, and they originated from both animals and humans. This indicated that drinking water from groundwater in a rural karst terrain is vulnerably polluted by ARB [77]. Zhang et al. (2014) found that the residue of 61 antibiotics (including quinolones, tetracyclines, and sulfonamides) in the Wenyu River basin was associated with quinolone-resistant *E. coli* [78]. The concentrations of AR *E. coli* in both an urban and a rural river ranged from 10 to 100 CFU/mL in New Zealand; ampicillin-resistant *E. coli* was the most common in both rivers [15].

Malema et al. investigated the prevalence and antibiotic resistance of pathogenic *E. coli* strains in harvested rainwater in South Africa by using PCR and disc-diffusion methods [79]. The result showed that *E. coli* isolates had the highest resistance to cephalosporin (76%), and 52% of isolates had multiple antibiotic resistance; all tested pathogenic *E. coli* isolates were sensitive to gentamicin, indicating that collected rainwater is not suitable for human drinking before treatment.

A total of 436 *E. coli* isolates with resistance to 17 antibiotics were found in water from representative interconnected sites (including streams, swallow caves, springs, and wells) in rural karst water systems in France [77]. Fakhr et al. (2016) investigated the contamination of drinking water by diarrheagenic AR *E. coli* in Zagazig City, northeastern Egypt [80]. The results showed that 16 *E. coli* strains were isolated from 300 potable water samples. All *E. coli* isolates were resistant to at least to one antibiotic, and 62.5% of *E. coli* displayed resistance to three or more antibiotics. *E. coli* exhibited high resistance to cefotaxime, tetracycline, and ampicillin (50–62.5%). High frequency of fecal contamination of drinking water indicates a high risk of diarrhea caused by AR *E. coli* [80]. Bong et al. (2022) investigated prevalence and diversity of AR *E. coli* from the anthropogenic-impacted Larut River and found that the concentration of AR *E. coli* carrying *tet* and *sul* genes was 4.1 × 10^3^–4.7 × 10^3^ CFU/mL in wastewater effluents, higher than in the river water; this indicated that *E. coli* is a key carrier of ARGs in freshwater river environments [74]. More effective measures should be taken to prevent and control the prevalence and risk of transmission of AR *E. coli* [79,81].

### 2.4. Modes of HGT in AR E. coli

Vertical gene transfer (VGT) and HGT are the two major mechanisms for the spread of AGRs in *E. coli* [25]. VGT is defined as a gene transferring from a parent to their offspring. Comparably, HGT, the process by which an organism passes genetic material to other cells rather than to its offspring, makes gene transfer more complicated. HGT is regarded as the most common mechanism of action and plays an important role in the rapid spread of antibiotic resistance in *E. coli* of animal origin, leading to the swift and wide transmission of ARGs between *E. coli* and other bacteria [29]. The transmission of AR *E. coli* by HGT depends mainly on the fact that resistant plasmids can be transmitted between different environments and hosts [27,82,83]. HGT mainly involves ARGs carried in MGEs, such as transposons, plasmids, and integrons [84,85]. The transfer of ARGs between *E. coli* of animal origin and other bacterial populations by HGT is governed mainly by three mechanism modes, including conjugation, transformation, and transduction [84,86,87]. Among them, conjugation is the primary mode of HGT, in which plasmids are transferred between donor and recipient cells that are in physical contact with one another through the mating pore pilus. The plasmids carrying ARGs such as *mcr-1* gene in *E. coli* from food animals were transferred to recipients of *E. coli* and *Salmonella* spp. of humans by conjugation, conferring resistance to polymyxin, apramycin, chloramphenicol, etc. [88,89]. Transformation refers to the transfer of short free DNA from the environment, which does not need living donor cells, and incorporation into recipient cells. Transduction is the process of transfer of DNA via bacteriophages [84,85]. Tetracyclines with sub-minimum inhibitory concentration (MIC) can promote conjugate transfer of *E. coli* plasmid (PR4) and accelerate HGT effects [90]. These mechanisms may cause the rapid evolution of *E. coli* because HGT can increase their fitness in the presence of antibiotics [91].

## 3. Disinfection of AR *E. coli* and Its ARGs

### 3.1. Traditional UV Treatment

**Figure 2:**
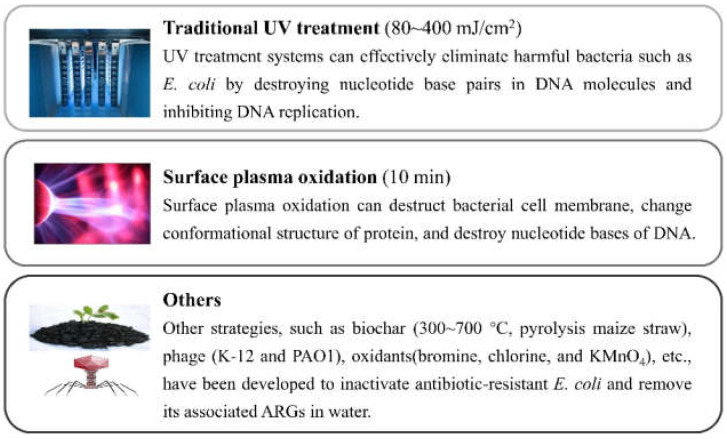
Strategies to eliminate AR *E. coli* and its ARGs.

UV treatment systems can effectively eliminate AR *E. coli* by destroying nucleotide base pairs in DNA molecules and inhibiting DNA replication (Figure 2) [92]. UV treatment does not produce any chemical by-products and is becoming more common in the treatment of AR *E. coli* and its ARGs in wastewater and drinking water.

Rizzo et al. (2013) evaluated the effects of UV radiation on AR *E. coli* from an urban wastewater treatment plant [93]. AR *E. coli* was inactivated after UV irradiation for 1 h (at a dose of 1.25 × 10^4^ μW s/cm^2^), while the traditional chlorination disinfection process (at a concentration of 2 mg/L) did not affect the antibiotic resistance of the investigated *E. coli* strains. Additionally, UV treatment did not change the amoxicillin and sulfamethoxazole resistance of *E. coli* (MIC > 256 or > 1024 μg/mL), but affected resistance to ciprofloxacin (MIC decreased by 33–50%). Conventional disinfection may be ineffective in the inactivation of ARB, which may be linked to UV levels [93].

It is vital to use suitable UV levels to eliminate AR *E. coli*. Pang et al. (2016) found that the UV dose of 40 mJ/cm^2^ led to a 5.5-log reduction in ampicillin-resistant *E. coli*, but *E. coli* was more resistant at lower UV doses (5–20 mJ/cm^2^) [94]. Zhang et al. (2017) isolated AR *E. coli* from a sewage treatment plant and evaluated the effects of UV irradiation on bacteria and their ARGs [34]. It was found that MDR *E. coli* was more resistant to UV disinfection at lower UV doses; the inactivation curves entered the tailing phase at a dose of 20 mJ/cm^2^, higher than that of antibiotic-sensitive *E. coli* (8 mJ/cm^2^). MDR *E. coli* was completely inactivated at a UV dose of 400 mJ/cm^2^. Moreover, ARGs (tetracycline- and sulfamethoxazole-resistant genes *tetA*, *tetB*, and *sul2*) of *E. coli* with 10^7^–10^8^ copies/mL were not effectively eliminated by UV disinfection; the reduced rates of relative abundance of ARGs reached 0.85–6 log after 80 mJ/cm^2^ UV irradiation [34]. O’Flaherty et al. (2018) detected the effects of different UV levels and lamp types on AR and antibiotic-sensitive *E. coli* in water treatments [57]. It was found that 7.5–8.4 mJ/cm^2^ UV lamp treatment led to a 6-log reduction in AR *E. coli*, higher that in antibiotic-sensitive *E. coli* (7.3–8.1 mJ/cm^2^); the UV levels were lower than the recommended level of 40 mJ/cm^2^ [57]. In general, UV treatment significantly reduced the number of viable AR *E. coli* cells, which is associated with the UV levels.

### 3.2. Surface Plasma Oxidation

Surface plasma oxidation can destroy the bacterial cell membrane, change the conformational structure of proteins, and destroy nucleotide bases of DNA (Figure 2) [73]. In recent years, more attention has been given to surface plasma in various bacteria inactivation due to its high efficiency, simple equipment, and no exogenous chemical residues [95].

Li et al. (2021) studied the effects of surface plasma on the elimination of AR *E. coli* and its ARGs from water environments [73]. The result showed that after plasma treatment for 10 min, 6.6 log *E. coli* was inactivated and the tetracycline, gentamicin, and amoxicillin resistance of *E. coli* significantly decreased, which may be associated with reactive oxygen and nitrogen species. Moreover, some ARGs (including *tet(C)*, *tet(W)*, *bla_TEM-1_*, and *aac(3)-II*) of *E. coli* were remarkably removed after the plasma treatment, and the conjugative transfer of ARGs (such as integron gene-*intI1*) was dramatically inhibited. This result highlights that plasma has potential application in removing AR *E. coli* and its ARGs from water environments [73].

Song et al. (2021) found that after surface plasma treatment for 10 min, 7.0 log AR *E. coli* in water was inactivated; its associated ARGs (including *tetC*, *tetW*, *bla_TEM-1_*, *aac(3)-II*, and *intI1*) decreased by 1.04–2.3 log copies, which may be attributed to oxidizing substances (such as H_2_O_2_, O_3_, NO_2_−, etc.) [31]. Additionally, the tetracycline, amoxicillin, and gentamicin resistance of *E. coli* decreased by 96.9–98.4%, and HGT of ARGs was suppressed by 63% after the plasma treatment. Overall, surface plasma treatment may be an effective method to remove AR *E. coli* and associated ARGs from water environments [31].

### 3.3. Others

Other strategies, such as biochar, phage, oxidants, etc., have been developed to inactivate AR *E. coli* and remove its associated ARGs from water (Figure 2) [30,31].

Combined biochar and polyvalent bacteriophage (phage) were used to inactivate AR *E. coli* and its ARGs in a soil–plant system [30]. The abundance of AR *E. coli* K-12 and its ARGs (such as *tetM*, *tetQ*, *tetW*, *ampC*, etc.) significantly decreased in the soil and in lettuce tissues following combined treatment for 63 d. A novel biotechnology used in this work provides insights into the targeted inactivation of AR *E. coli* and its ARGs, therein reducing their dispersion risks in the soil–plant–human system [30].

Disinfection efficiencies of AR *E. coli* and the sulfonamide-resistant gene *sul1* were carried out in sterilized pond water by three fishery oxidants (bromine, chlorine, and KMnO_4_) [32]. After treatment with the dosages of 5–15 mg/L of the three oxidants, AR *E. coli* was completely inactivated and *sul1* was efficiently removed. Chlorine had a higher ability to eliminate AR *E. coli* than bromine and KMnO_4_; chlorine and bromine had moderate removal efficiency of *sul1*. The results indicated that oxidative treatments may help with practical disinfection to prevent the spread of ARB and their ARGs in aquaculture environments [32]. Additionally, other biological or chemical methods (such as peracetic acid) effectively eliminated AR *E. coli* (<10^2^ CFU/mL) in wastewater [14,71].

## 4. Alternatives to Antibiotics to Combat AR *E. coli* from the Farm Animal-Associated Sources

Some strategies have been taken to minimize antibiotic resistance, including the complete restriction of antibiotics used in food animals, the prudent use of conventional antibiotics, the development of alternatives to antibiotics, etc. [96,97,98]. At present, over 128 countries, including in Europe (such as Sweden in 1986, Denmark and Norway in 1995, Germany in 1996, etc.) and Asia (such as Japan in 2008, Korea in 2011, Vietnam in 2017, China in 2020, etc.) and the United States (in 2017), have taken action to regulate the use of antibiotics in food animals by banning, restricting, and reducing the amount of growth-promoting antibiotics in animal feed [99,100]. Moreover, some probiotics, herbal medicines, and AMPs have been developed to replace the use of antibiotics.

### 4.1. Probiotics/Prebiotics

**Figure 3:**
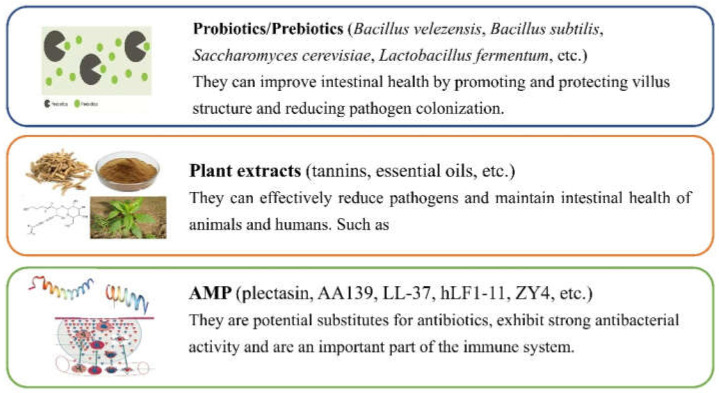
Alternatives to antibiotics to fight against AR *E. coli*.

Probiotics are one of the potential alternatives to antibiotics due to their modulation of the gut microbiota and enhancement of growth performance in animals (Figure 3). 

*Bacillus subtilis* can enhance the immune response and it maintains the balance of intestinal flora; it increases the abundance of *lactic acid bacteria* and *bifidobacterial*, and reduces *coliform bacteria* and *Clostridium perfringens*. Moreover, short-chain fatty acids produced by *B. subtilis* can enhance intestinal health [101]. The *B. velezensis* ZBG17 strain exhibited high stability towards gastroenteric fluid in animal guts [102]. *B. velezensis* ZBG17 completely inhibited bacterial pathogens such as AR *E. coli* and *Salmonella enteritidis* within 6–8 h, and significantly improved the feed efficiency and humoral immune response in broilers. This highlights that *B. velezensis* ZBG17 is a prospective alternative to antibiotics in broiler production [102]. Bilal et al. found that the growth of *E. coli* O78 was inhibited by feeding 1.5 g/kg *Saccharomyces cerevisiae* (2 × 10^6^ CFU/g) and *Lactobacillus fermentum* (1 × 10^7^ CFU/g) that serves as a prebiotic and probiotic [103]. The supplement of prebiotics and probiotics into animal feed can improve animal welfare and productivity by promoting and protecting the villus structure and reducing pathogen colonization, such as *E. coli* in the poultry intestinal tract [103]. The data provide a new concept of a commercially viable alternative to antibiotics in the broiler feed industry.

### 4.2. Plant Extracts

Botanicals play an important role in bacteriostasis and they are being tested as alternatives to antibiotics due to their positive effects on coliform diarrhea caused by AR *E. coli* (Figure 3) [37].

Dell’Anno et al. investigated the effects of quebracho/chestnut tannins (0.75%) extracts, leonardite (0.25%), and tributyrin (0.2%) on porcine colibacillosis [104]. It was found that the combination of plant extracts increased the ratio of *Lactobacillus* to coliform in feces and reduced the incidence of diarrhea in weaned piglets [104]. This indicates that plant extracts are regarded as potential alternatives to antibiotics to maintain swine health and performance. Plant extract-concentrated tannin showed efficient antibacterial activity toward avian AR *E. coli* in vitro [105].

Recent studies showed that various plant essential oils (including terpenoids and phenylpropenes) have potent antimicrobial activity and could effectively reduce pathogens such as *E. coli* [106,107,108]. The counts of precaecal *E. coli* in broilers significantly reduced after the supplement of cinnamon bark oils with 300–600 mg/kg of diet [109]. Meanwhile, essential oils can maintain intestinal health and promote growth, indicating that they can be green alternatives to antibiotics in animal production to combat AR *E. coli* [108].

### 4.3. AMPs

It has been demonstrated that a few AMPs (such as plectasin, AA139, LL-37, hLF1-11, ZY4, etc.) in different phases of clinical trials (phase I–III) against ARB are more effective than conventional antibiotics due to their low resistance (Figure 3) [110,111,112,113]. AMPs have potent antibacterial activity, increasing the permeability of the bacterial inner and outer membrane, destroying the cell membrane, and promoting the leakage of intracellular substance, which may result in low resistance [114]. Bacteriocin is a kind of peptide or precursor peptide with potent antibacterial activity against *E. coli* [115]. Several Ib-M peptides (with the length of 20 amino acids) displayed potent activity against aminoglycoside-resistant *E. coli* O157:H7 AC188 with the MIC value of 1.6–6.3 μM; after exposure to 1 × MIC Ib-M peptides for 4 h, the population of *E. coli* reduced by more than 95%, indicating potential promising molecules for the development of new alternatives to antibiotics to combat AR *E. coli* [116].

Microcins produced by Gram-negative bacteria have been regarded as potential alternatives to antibiotics [117]. Lu et al. (2019) demonstrated that microcin PDI (MccPDI) generated by *E. coli* could inhibit MDR *E. coli* and *Shigella* isolates by the disruption of the bacterial membrane, indicating its potential as an alternative to antibiotics [118].

Other AMPs, such as dermaseptins, cathelicidin-OH-CATH30, and magainin-PGLa, have provided alternative therapies against MDR bacteria, including *E. coli* [41,42,43]. Generally, although several AMPs provide a promising revenue to reduce ARB, the road ahead is still long due to high production costs, poor stability in vivo, and other side effects [119,120].

## 5. Conclusions

The increasing use of antibiotics in farm animals has led to the spread of AR *E. coli* and its ARGs. Here, we have reviewed the prevalence of AR *E. coli* of farm animal origin. Disinfection methods (such as UV treatment, surface plasma oxidation, biochar, oxidants, etc.) and a few alternatives to antibiotics (including probiotics, plant extracts, and AMPs) to combat AR *E. coli* were highlighted, which may help us address the issues of bacterial antibiotic resistance from the farm animal-associated sources.

## Figures and Tables

**Table 1:** AR *E. coli* and its ARGs in food animals and products (2016–2022).

Sources	Resistance to Antibiotics	ARGs	Location	References
Eggs	Florophenicol	NN	Slovenia	[45]
Turkey	ampicillin, ampicillin–sulbactam, cefazolin, and tetracycline	*uidA*	America	[46]
Broiler chicken	Colistin, ampicillin, tetracycline, and chloramphenicol	*strA*, *strB*, and *bla_TEM-135_*	Germany	[49]
Fresh seafood in retail markets	Cephalosporins cefotaxime, cefpodoxime, ceftazidime, imipenem, cefoxitin, and meropenem	*bla_CTX-M_*, *bla_SHV_*, *bla_TEM_*, *bla_OXA_*, *bla_NDM_*, and *bla_VIM_*	India	[3]
Raw beef	Colistin and cefotaxime	*mcr-1* and *E_CTX-M-28_*	Egypt	[55]
Ready-to-eat beef products	Colistin and cefotaxime	*mcr-1* and *bla_TEM-116_*	Egypt	[55]
Milk	Amoxicillin–clavulanic acid, ampicillin, ceftriaxone, kanamycin, streptomycin, trimethoprim sulfamethoxazole, and vancomycin	*bla_CTX-M_*, *bla_TEM-1_*, *bla_NDM-1_*, *bla_OXA-48_*, *bla_VIM_*, and *bla_SHV_*	Pakistan	[56]
Malaysian broiler chicken	Carbapenem and colistin	*mcr-1*, *bla_TEM-52_*, *bla_NDM_*, *bla_OXA-48_*, and *bla_IMP_*	Malaysia	[52]
Vietnam broiler chicken	Colistin	*bla_CTX-M_*, *mcr-1*, *bla_TEM_*, and *bla_CMY-2_*	Vietnam	[53]
Retail mutton	Trimethoprim, sulfamethoxazole, aminoglycosides, quinolones, and nitrofurantoin	*bla_CTX-M_*, *bla_TEM_*, and *bla_SHV_*	Egypt	[54]

**Table 2:** AR *E. coli* and its ARGs in animal waste (2016–2022).

Sources	Resistance to Antibiotics	ARGs	Strategies	Location	References
Veal calf	Tetracycline, ampicillin, sulfonamides, streptomycin, and trimethoprim	*intI1 and intI2*	NN	French	[60]
South African herbivores	Gentamicin, tobramycin, ceftazidime, and aztreonam	NN	Antibiotics used in human medicine should be avoided in veterinary medicine	South Africa	[61]
Free-grazing food animals	Ampicillin, ceftriaxone, chloramphenicol, gentamicin, streptomycin, sulfonamide, tetracycline, etc.	*dfrA*, *orfF*, *aadA*, *sul1*, and *qacEΔ1*	NN	China	[62]
Meat duck deep litter	Ceftiofur, enrofloxacin, ofloxacin, and gentamicin	NN	The deep litter should be treated with appropriate antibiotic resistant bacteria	China	[8]
Dog	Cefazolin and fluoroquinolone	*bla_SHV_*, *bla_TEM_*, *bla_OXA_*, and *bla_CTX-M_*	NN	Taiwan, China	[2]
Intensively produced poultry	Ampicillin, tetracycline, nalidixic acid, trimethoprim-sulfamethoxazole, and chloramphenicol	*bla_CTX-M_*, *sul1*, *tetA*, and *tetB*	NN	South Africa	[23]
Open pig farm	Chlortetracycline, tetracycline, tilmicosin, amoxicillin, and doxycycline	NN	Legislation to clarify the boundary between antibiotics for human use and antibiotics for veterinary use	Thailand	[63]
Deer and Pigs	Ciprofloxacin and nalidixic acid	*qnrS* and *qnrB*	NN	Germany	[64]
Cow farm	Tetracycline	*tetA*, *tetB*, and *tetM*	Veterinarian supervisor, administration history for all individuals, and the rearing environment is strictly managed	Japan	[65]

NN: no data.

## Data Availability

The data are contained within the article.
